# Cyclin-Dependent Kinase 4/6 Inhibitor (Palbociclib) Induced Aplastic Anemia in a Patient with Metastatic Breast Cancer

**DOI:** 10.1155/2018/9249506

**Published:** 2018-12-13

**Authors:** Stanley Madu Nwabudike, Camille V. Edwards, Oladimeji Akinboro, Kathryn Quinn, Shayna Sarosiek, Naomi Ko

**Affiliations:** Section of Hematology and Medical Oncology, Department of Medicine, Boston Medical Center, Boston, Massachusetts, USA

## Abstract

Breast cancer is the most common cancer diagnosed in women worldwide. Over the years, breast cancer treatment has undergone revolutionary changes especially for women with hormone receptor positive metastatic disease. As a result, women are living longer with their disease, particularly in developed countries. The use of cyclin-dependent kinase (CDK) 4/6 inhibitors with antiestrogen therapy is a relatively new therapeutic option which has been shown to improve progression-free survival. Hematologic adverse events, most frequently neutropenia, are well-known side effects of CDK 4/6 inhibitors. However, to our knowledge, aplastic anemia has never been reported. We report a case of aplastic anemia in a patient with metastatic breast cancer treated with palbociclib after multiple prior lines of therapy.

## 1. Introduction

In 2018, breast cancer accounted for about 15% of new cancer diagnoses in the United States, with an estimated 268,670 new cases and 41,400 deaths (7% of all cancer deaths). It remains the most common cancer in women worldwide and hormone receptor (HR+) breast cancer constitutes about 80% of cases [1, 2]. In the last 3 decades, the survival has improved significantly, with approximately 90% of patients with early stage breast cancer alive 5 years after diagnosis [3]. With longer survival of breast cancer patients and new therapeutic approaches, previously unknown adverse treatment effects may be discovered. Documenting and managing these adverse effects are crucial in caring for these patients.

Cyclin-dependent kinase (CDK) 4/6 pathways are overactive in several cancers, and inhibition of this pathway offers a relatively novel therapeutic approach to breast cancer management by activation of the tumor suppressor retinoblastoma (RB) gene leading to cell cycle arrest and cellular senescence. This prevents or controls the movement of dividing cells from G1 to S phase of the cell cycle, during which DNA synthesis usually occurs [4, 5]. Palbociclib is a CDK 4/6 inhibitor approved for the treatment of postmenopausal women with estrogen receptor (ER) positive metastatic breast cancer in combination with an antiestrogen [6–8]. The most common side effects of the CDK 4/6 inhibitors include hematologic toxicity, such as neutropenia and thrombocytopenia, and gastrointestinal toxicity.

Aplastic anemia (AA) is a rare heterogenous disease characterized by pancytopenia due to bone marrow aplasia or hypoplasia [9]. It may be idiopathic, but the majority of cases are secondary to an insult to the bone marrow which can occur with medications, chemical exposures, viral infections, or immune disorders with subsequent autoimmune destruction of hematopoietic stem cells [9, 10]. The incidence is estimated at approximately two per million per year in western countries and is more common in Asian countries [10].

We report a case of a postmenopausal woman heavily treated for metastatic hormone receptor positive breast cancer who developed AA during treatment with palbociclib.

## 2. Case

The case is of a 67-year-old woman who was initially diagnosed at age 36 with early stage ER+ left breast invasive ductal carcinoma, treated with breast-conserving surgery followed by adjuvant radiation therapy. Unfortunately, she developed recurrence in the thoracic spine 16 years later which was treated with surgery and postoperative thoracic spine radiation followed by hormone therapy (letrozole and fulvestrant) and cytotoxic chemotherapy (capecitabine) over a four-year period. She recurred in the cervical spine and was treated with cervical spine radiation followed by liposomal doxorubicin and then paclitaxel. Since the initial occurrence of bone metastases, she was also treated with a bone modifying agent, initially pamidronate and then switched to denosumab due to worsening renal function.

Ten years after this, her disease progressed with multiple new bone metastases on PET/CT scan, and a decision was made to switch to palbociclib in combination with fulvestrant. Her other medical conditions included well-controlled type 2 diabetes mellitus, hypertension, obstructive sleep apnea, stage 3 chronic kidney disease, and recurrent urinary tract infections. Her baseline complete blood count (CBC) at start of treatment with palbociclib was notable for mild anemia with hemoglobin (Hb) range 8–9.5 g/dL (ref. 11.8–16 g/dL) but normal white blood cell (WBC) count 8.2 × 10^9^/L (ref. 4–11 × 10^9^/L) with normal differential and platelet counts 345 × 10^9^/L (ref. 150–400 × 10^9^/L). The initial palbociclib dose was 125 mg daily on days 1–21 of a 28-day cycle in combination with monthly fulvestrant 500 mg intramuscularly.

On routine clinic visit during cycle 3, week 2 of treatment, she was found to have new pancytopenia (WBC 3.9 × 10^9^/L (with neutropenia and monocytopenia), Hb 6.9 g/dL, and platelet count 117 × 10^9^/L). On examination, she had pallor but no icterus, hepatosplenomegaly, nor clinically palpable lymph nodes. Extensive review of her medication list did not reveal potential culprits for her pancytopenia. Routine blood tests for other causes of pancytopenia including vitamin B12, iron studies, folate, thyroid studies, hepatitis C, and HIV were normal. Serum erythropoietin level was appropriately elevated at 53.6 mIU/ml (ref. 2.6–18.5 mIU/ml). She was transfused with 2 units of packed red blood cells (PRBCs), and palbociclib was continued with dose modification.

**Figure 1:**
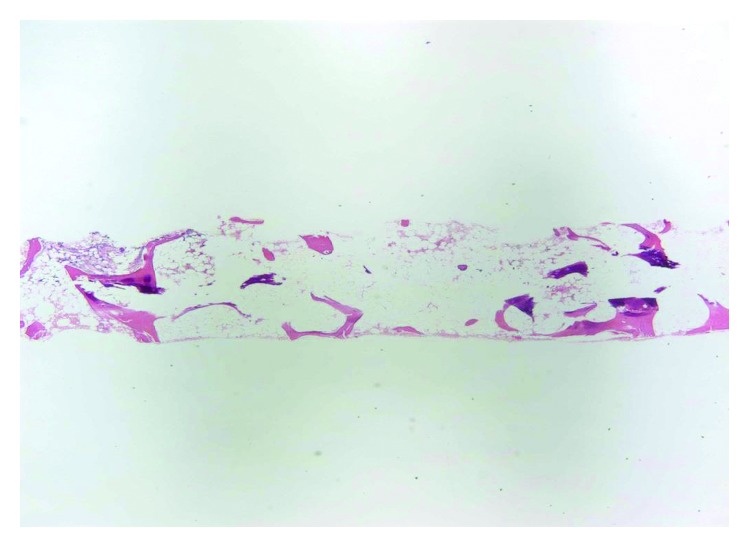
Hematoxylin and eosin stain at 20× magnification showing a markedly hypoplastic, near aplastic bone marrow.

**Figure 2:**
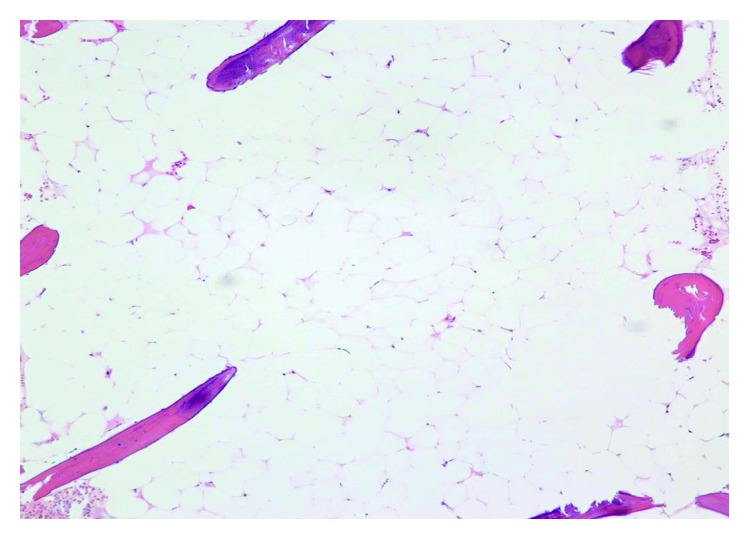
Hematoxylin and eosin stain at 100× magnification showing most of the bone marrow showing aplasia with absence of marrow elements.

**Figure 3:**
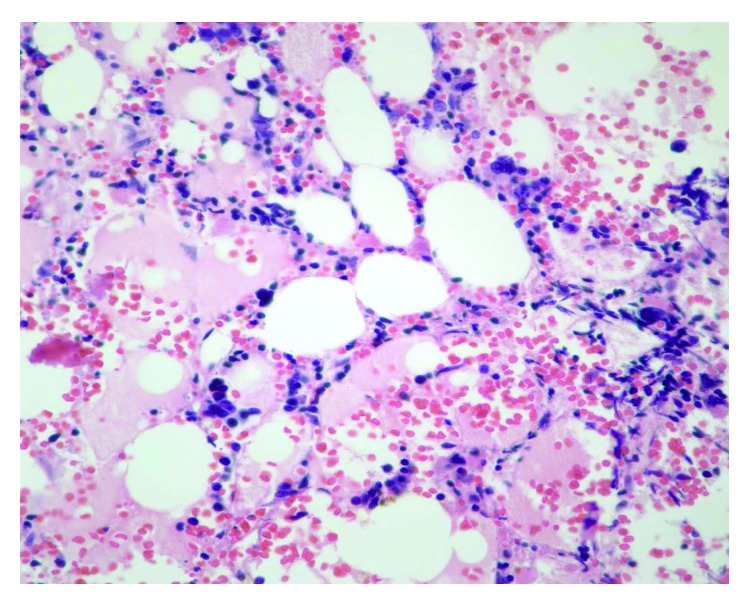
Hematoxylin and eosin stain at 400× magnification of a rare focus showing residual trilineage hematopoiesis, in an otherwise near aplastic bone marrow.

Interval PET/CT scan done after cycle 3 showed stable bone disease with no new bone or visceral lesions. Tumor markers remained elevated but stable. Pancytopenia persisted with worsening leukopenia and thrombocytopenia (WBC dropped to 2.2 × 10^9^/L with neutropenia and platelets 24 × 10^9^/L). Reticulocyte count was inappropriately normal at 0.9% (absolute reticulocyte counts 30,000 cells/microliter). She continued to require red cell transfusions and developed bleeding gums with minimal trauma. Peripheral blood smear revealed mild anisocytosis with nucleated red cells (12 nucleated RBCs per 100 leukocytes), leukopenia with no dysplastic changes or blasts, and thrombocytopenia without clumping or giant platelets. Given these findings on peripheral smear and prolonged cytopenia, a bone marrow biopsy was performed to exclude a bone marrow infiltrative or leukemic process. Bone marrow biopsy/aspirate showed features consistent with severe aplastic anemia (few pockets of trilineage hematopoiesis accounting for about 2% of total cellularity and 98% fat) (Figures 1–3). There was no evidence of marrow metastases, dysplastic features, or myelophthisis. Normal flow cytometry on bone marrow aspirate ruled out a leukemic process. Cytogenetic testing was normal (46XX in all 20 mitotic cells analyzed). Next Generation Sequencing for 42 somatic genetic mutations associated with myeloid disorders and neoplasms including myelodysplastic syndrome was negative.

Due to continued red cell transfusion dependence and persistent pancytopenia despite dose interruptions, palbociclib was discontinued after a total of 4 months. After discontinuation of palbociclib, her cytopenias improved significantly despite treatment of her progressing breast cancer with multiple other therapies. Her WBCs and platelets have now normalized, and her transfusion requirements are markedly decreased (from an average 1 unit red cells every 2 weeks to 1 unit every 6 to 8 weeks). At the time of this report, she had not received red cell transfusion in 2 months. The most recent CBC, 7 months after palbociclib was discontinued, is as follows: WBC 10.4  × 10^9^/L (normal WBC differential), Hb 8.7 g/dL, and platelets 256 × 10^9^/L. Interestingly, her mean corpuscular volume (MCV) at the initiation of palbociclib was 83 fL and went up to 95 fL by the 4^th^ month and trended downwards after discontinuation.

Given the recovery in her blood counts and clinical improvement after discontinuing palbociclib, a bone marrow biopsy was not repeated as this is unlikely to alter management strategy. She is followed closely in the medical oncology clinic.

## 3. Discussion

Palbociclib, a reversible small-molecule CDK 4/6 inhibitor, was approved based on the PALOMA clinical trials [6] for the treatment of postmenopausal women with ER-positive metastatic breast cancer in combination with an antiestrogen [7, 8]. When used with aromatase inhibitors or fulvestrant, it was shown to improve PFS. In the clinical trials, the most common adverse effect of palbociclib is neutropenia (up to 80%) and less common hematological adverse effects include anemia (24%) and thrombocytopenia (16%) [11].

Aplastic anemia (AA) manifests with pancytopenia and bone marrow aplasia/hypoplasia which may be due to immune causes, infections, or toxins such as medications [9]. Irrespective of the etiology, loss or defect of hematopoietic stem cells (HSCs); which are capable of self-renewal and differentiation into mature blood cells, is the key pathogenic mechanism for AA. This is accompanied by autoimmune destruction of these defective stem cells which ultimately leads to pancytopenia. It has been suggested that the severity of AA may depend on the strength of immune response; more robust or vigorous immune reactions lead to more severe forms of AA [12, 13]. Treatment of AA depends on the severity based on the Camitta criteria [14, 15] ranging from supportive care with transfusions for nonsevere cases, to use of immunosuppressive agents (with or without eltrombopag) and hematopoietic stem cell transplant for very severe cases. Additionally, the offending agent must always be removed where possible. Our patient had the nonsevere form (absolute neutrophil count >0.5 × 10^9^/L and platelet count >20 × 10^9^/L), so she was treated with removal of the offending agent and simple red cell transfusions.

Another close differential diagnosis is hypoplastic myelodysplastic syndrome (MDS) which may be very difficult or almost impossible to distinguish from AA. MDS, with reversible macrocytic anemia, attributed to palbociclib has been reported recently. Three cases of reversible MDS were observed and reported by Anampa et al. [16]. These patients had dysplastic changes in their peripheral blood, and they all had bone marrow aspirates/biopsies which also showed dysplastic changes in erythroid precursors and megakaryocytes with bone marrow cellularity of about 10%. An additional cohort study showed development of macrocytosis and red cell dysplasia in 34 patients treated with palbociclib, typically after 4 cycles. The dysplastic changes were reversible on discontinuation of palbociclib [16]. Although our patient developed worsening anemia with mildly elevated MCV, dysplastic changes were not seen in the bone marrow aspirate. In addition, cytogenetic and molecular studies were negative for characteristic changes associated with MDS. Therefore, our patient's cytopenias were more likely related to AA and not hypoplastic MDS.

There is paucity of data on the manifestations of anemia induced by palbociclib. However, aplastic anemia is not a known or reported adverse effect of palbociclib. Unlike cytotoxic chemotherapy, palbociclib induces bone marrow toxicity by reversible hematopoietic non-lineage-specific cell cycle arrest at G1-S phase and not by apoptosis or DNA damage. Therefore, the effect can typically be reversed on withdrawing the drug [5]. We postulate that the AA observed in our patient may be due to HSC senescence with consequent immune destruction and less likely, but possibly, an idiosyncratic reaction.

To the best of our knowledge, this is the first reported case of aplastic anemia due to palbociclib in treatment of breast cancer. The reason for the lack of reported cases may be due to the multitude of causes that may contribute to cytopenias in a patient with advanced breast cancer. This was also true in our patient who had received multiple lines of prior therapy, including an anthracycline, paclitaxel, and radiation to thoracic and cervical spine, which may have predisposed her to pancytopenia due to poor bone marrow reserve. Her blood counts had recovered after each of those therapies and recovered with discontinuation of the CDK 4/6 inhibitor, suggesting palbociclib as the likely etiology for AA. Ideally, a repeat bone marrow biopsy showing reversal of marrow aplasia would confirm our hypothesis, but an improved complete blood count and reduced frequency of red cell transfusion could serve as a surrogate marker for bone marrow recovery.

## 4. Conclusion

With the expanding use of CDK 4/6 inhibitors in breast cancer treatment, more hematologic adverse effects may be observed [17]. Aplastic anemia has never been reported with CDK 4/6 inhibitors, and to the best of our knowledge, our case is the first reported. AA developed about 3 months after initiation of palbociclib and improved with discontinuation of drug. Given the relative novelty of these agents in breast cancer treatment, it is important to note this potentially serious adverse effect and have a low index of suspicion in patients receiving palbociclib, especially those previously heavily treated for their disease. Additional clinical research is needed to determine the exact etiology of hematologic toxicity of palbociclib and to determine which patients are at risk for these toxicities.
